# Long-Term Dietary Restriction Leads to Development of Alternative Fighting Strategies

**DOI:** 10.3389/fnbeh.2020.599676

**Published:** 2021-01-14

**Authors:** Jeanne Legros, Grace Tang, Jacques Gautrais, Maria Paz Fernandez, Séverine Trannoy

**Affiliations:** ^1^Research Center on Animal Cognition (CRCA), Center for Integrative Biology, Toulouse University, CNRS, UPS, Toulouse, France; ^2^Department of Neuroscience and Behavior, Barnard College of Columbia University, New York, NY, United States

**Keywords:** fighting strategies, adaptation, dietary restriction, social rank, *Drosophila melanogaster*, aggression

## Abstract

In competition for food, mates and territory, most animal species display aggressive behavior through visual threats and/or physical attacks. Such naturally-complex social behaviors have been shaped by evolution. Environmental pressure, such as the one imposed by dietary regimes, forces animals to adapt to specific conditions and ultimately to develop alternative behavioral strategies. The quality of the food resource during contests influence animals' aggression levels. However, little is known regarding the effects of a long-term dietary restriction-based environmental pressure on the development of alternative fighting strategies. To address this, we employed two lines of the wild-type *Drosophila melanogaster* Canton-S (CS) which originated from the same population but raised under two distinct diets for years. One diet contained both proteins and sugar, while the second one was sugar-free. We set up male-male aggression assays using both CS lines and found differences in aggression levels and the fighting strategies employed to establish dominance relationships. CS males raised on a sugar-containing diet started fights with a physical attack and employed a high number of lunges for establishing dominance but displayed few wing threats throughout the fight. In contrast, the sugar-free-raised males favored wing threats as an initial aggressive demonstration and used fewer lunges to establish dominance, but displayed a higher number of wing threats. This study demonstrates that fruit flies that have been raised under different dietary conditions have adapted their patterns of aggressive behavior and developed distinct fighting strategies: one favoring physical attacks, while the other one favoring visual threats.

## Introduction

Aggression is an innate and complex social behavior observed throughout the animal kingdom that takes different forms: threat displays, physical approaches, chases, and physical attacks. Multiple aggressive interactions with high-intensity physical attacks among members of a social group lead to the formation of hierarchies (Chase and Seitz, 2011). Once established, a stable social hierarchy structures the group, decreasing future aggressive interactions among members. Therefore, animals tend to employ the best fighting strategy to reach a short- or long-lasting social consensus (Holekamp and Strauss, 2016).

Although an innate behavior, aggression contains adaptive features crucial for animals living in constantly changing environments (Reichert and Quinn, 2017). Two types of behavioral plasticity are related to environmental changes: (i) short-term with changes in color, size, or locomotor activity in response to novel but predictable environmental modifications, and (ii) long-term plasticity involving development of alternative and irreversible behavioral phenotypes in response to environmental pressure (Brockmann, 2001). Dietary-restriction is one example of driving force exerted on animals to adapt to limited conditions and ultimately to develop alternative behavioral strategies (Han and Dingemanse, 2015; Zhang et al., 2019). Dietary-restriction is known to induce behavioral changes and reduce the reproductive yield (Adler et al., 2013), affect flight endurance in insects (Nguyen, 2008), and extend lifespan in a wide range of animal species (Nakagawa et al., 2012). Diet is also known to modulate social behaviors, including aggression (Wallner, 2009). In humans, eating disorders enhance the frequency of aggressive behavioral manifestations (Truglia et al., 2006). Regarding the effect of different macronutrients on social behaviors, it has been shown that male rats fed with carbohydrates present a higher rate of fighting behavior and anxiety-like behavior (Hanstock et al., 2004), while Argentinian ants show lower level of aggression when deprived of sucrose (Grover et al., 2007). Moreover, Gottingen minipigs subjected to a high fat/low carbohydrate regime present a decrease in aggressive behavior (Haagensen et al., 2014). However, little is known about how the fighting strategies developed by animals are influenced by dietary regimes.

A variety of studies on invertebrates showed that aggressive behavior is modulated by genetic factors (Dierick and Greenspan, 2006), environmental conditions (Rittschof and Robinson, 2013; Rillich et al., 2019), social influences (Kilgour et al., 2019; Balsam and Stevenson, 2020), sex (Benelli et al., 2015), and previous experiences (Goubault and Decuigniere, 2012; Rose et al., 2017). Indeed, previous victory and defeat induce behavioral plasticity in the form of winner and loser effects (previous victory/defeat increase the probability of winning/losing subsequent fight) (Hsu et al., 2006). *Drosophila melanogaster* represents an attractive model to study the environmental influences on aggressive behaviors, dominance relationships and the development of alternative fighting strategies. In competition for food, mates, and territory, fruit flie*s* exhibit a series of stereotypical sex-specific aggressive patterns, but only males establish dominance between competitors using the male-specific lunge behavior (Chen et al., 2002; Nilsen et al., 2004). For this reason, our study focuses on males' aggressive behaviors. Flies also display visual threats, but their exact function remains debated: are they “bluffs” or “honest” signals? On one hand, wing threats displayed throughout the fight might reinforce the functions of lunges in escalating fights. On the other hand, they might serve independent functions. Yet, wing threats are not always considered a crucial element of the fighting strategy when analyzing Drosophila aggression. Nevertheless, the observation that 3 neurons promote threat displays without interfering with other types of agonistic behavior, supports the notion that lunges and wing threats are independent patterns controlled by distinct sets of neurons (Duistermars et al., 2018).

Studies have shown a correlation between male aggression levels and foraging-related behavior (Wang and Sokolowski, 2017), high fat dietary regimes (Meichtry et al., 2020), and the food value available during aggression assays (Lim et al., 2014). However, it remains to be determined how dietary regimes influence aggressive patterns and the development of fighting strategies. In addition, it is still unclear whether wing threats are an integral part of the fighting strategy used by flies to form and maintain dominance.

Here, using two lines of the wild-type Drosophila Canton-S (CS) that originated from the same population but raised under two distinct diets for about 10 years, we found that flies exhibited behavioral plasticity in response to distinct environmental conditions, leading to two different fighting strategies. Our results indicate that males from the line raised on a sugar-containing diet started fights with lunges and escalated fights quickly. Moreover, dominant individuals used lunges to establish and maintain dominance relationships. On the contrary, males raised in the sugar-free diet started fights either with lunges or wing threats and escalated fights to establish dominance with fewer lunges. In this case, dominants displayed threats to maintain their social rank, avoiding using higher-intensity patterns such as lunges. The differences in aggression levels based on lunges and fighting strategies could not be reversed by switching diets. Our data highlights a potential link between aggression levels, the development of alternative fighting strategies and dietary regimes.

## Materials and Methods

### Flies Stocks

Flies were raised at 25°C under a 12 h:12 h light/dark cycle (LD = 8:30 a.m.−8:30 p.m.). Two populations of *D. melanogaster* were used in this study: CS_A_ (from Edward Kravitz's laboratory at Harvard Medical School, Boston, USA) and CS_B_ (from Guillaume Isabel's laboratory at the Research Center of Animal Cognition CRCA, Toulouse, France). These CS lines were raised from 2010 to 2019 on standard medium, respectively: 52% cornmeal, 28% yeast, 121% sugar, 15% agar, 20% Moldex, and 70% corn flour, 70% yeast, 0% sugar, 9% agar, 20% Moldex. Since we started the study on October 2019, CS_A_ and _B_ were maintained on a medium labeled the *sugar-containing* diet that was composed of: 74% corn flour, 28%yeast, 40% sugar, 8% agar, 20% Moldex (to match as best as possible the recipe from HMS for raising CS_A_), and on a medium called *sugar-free* that was composed of: 70% corn flour, 70% yeast, 0% sugar, 9% agar, 20% Moldex (the same recipe used at the CRCA for raising CS_B_) (Supplementary Table 1 summarizes the composition of the two diets used, and Supplementary Figure 1 the experimental design).

### Experimental Chamber

The experimental setup used in this study to examine social behaviors has already been described (Trannoy et al., 2015). Briefly, a divider was inserted through the top of the arenas (22 mm diameter x 16 mm height) which contained a food cup (13 mm diameter x 6 mm height) separating them into two equal sizes. Flies were then inserted on each side of the arenas by negative geotaxis, so they can acclimate without interacting with each other. Behavioral experiments start once the separator was removed allowing flies to interact together.

### Behavioral Assays

On day 0, late stage male pupae were sexed and socially isolated in vials containing 1 ml of either sugar-containing or sugar-free diet, for 7 days, under 25°C 12 h:12 h LD cycles as described above. On day 5, flies were anesthetized with CO_2_ to apply a dot of paint on the dorsal thorax of flies for identification purposes. At day 7, behavioral experiments were performed between Zeitgeber time zero (ZT0, right after the lights on transition) and continued for up to 3 h (ZT3). During the maintaining and isolation phases, light conditions were constant (12 h:12 h L/D cycle = 8:30 a.m. to 8:30 p.m.). All behavioral experiments were performed during the first 3 h after the lights on transition (ZT0 to ZT3).

#### Aggression Assays

Two males from the same CS line were paired in each chamber with a food cup containing fresh fly food (either sugar-containing or sugar-free diet) with a drop of yeast paste on the surface. We scored all aggressive patterns that happened on the food cup and for 10 min after the time of the first lunge. If no lunges were observed for 15 min after t0 (time when the divider was removed from the arenas), we stopped the scoring. The latencies to lunge, wing threat (WT) represent the time between the first meeting and the first lunge and the time between t0 and the first WT. The latency to dominance is the time between the first meeting and the time to dominance. Time to dominance was determined when the putative loser retreats from the food cup three times after having received lunges from the other (Trannoy et al., 2016). Fight outcomes were either (i) no fight: when 0 lunges were observed, (ii) draw: when lunges were observed but were not sufficient to induce dominance or because of retaliation, or (iii) dominance: when dominance has been established between competitors during the 15 min after t0 of observation (Supplementary Table 2 summarizes the behavioral parameters used to score aggressive behavior).

#### Courtship Assays

One sexually mature male and one virgin female (both 7-days old) from the same CS line were inserted into each side of a behavioral chamber in the absence of a food resource. Courtship Vigor Index (CVI) was calculated as the fraction of time that males spent courting the female (including tapping, wing extension and vibration, chasing and attempted copulation) during a 10-min period after the first courtship behavior. The latencies to court and to copulate were the times between the first meeting and the first courtship behavior or the initiation of copulation, respectively.

#### Activity and Sleep Assays

Locomotor activity and sleep profiles were recorded using DAM2 *Drosophila* Activity Monitors (Trikinetics, Waltham, MA). Three-to five-day old males from CS lines were placed individually in Trikinetics capillary tubes containing either their “respective” or “switched” food. Flies were entrained to 12 h:12 h LD cycles for 5 days at a constant temperature of 25°C. Activity counts were collected in 1-min bins that were subsequently summed into 30-min bins for the time-series analysis of locomotor activity. Activity levels were normalized for individual flies by setting the average activity level for all 30-min bins across days 3–5 equal to 1.0. Population profiles were then averaged into a single representative 24-h day, displayed as histograms. For sleep quantifications, beam-crossings were also collected in 1-min bins. A sleep bout was defined as a period of inactivity of at least 5-min (Hendricks et al., 2000). Sleep plots represent averaged population sleep profiles and were obtained by averaging the sleep data over days 3–5 of the LD cycle, displayed as line plots.

### Statistical Analysis

**Figure 1:**
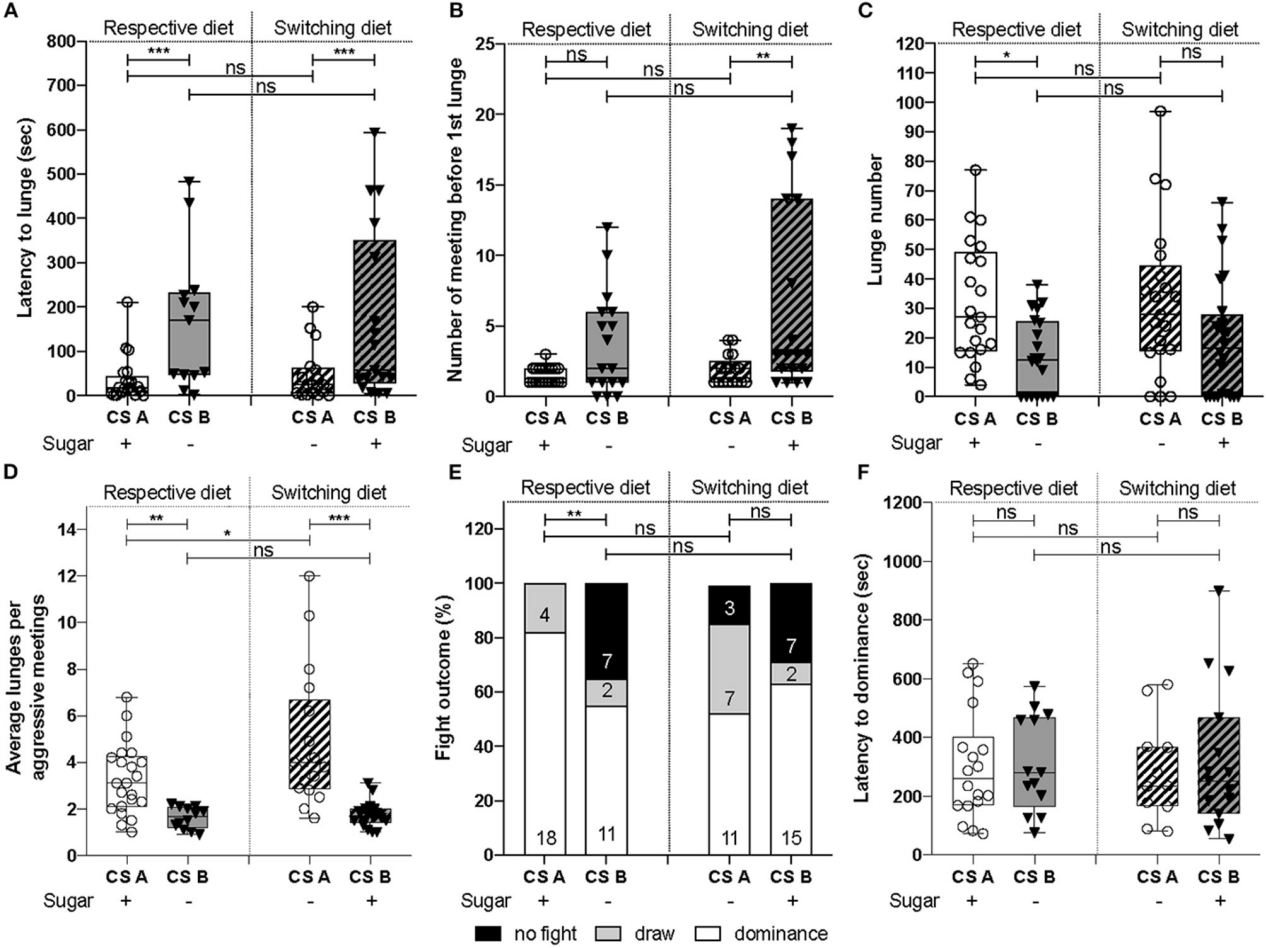
Dietary sugar content leads to different aggression levels. **(A)** CS_A_ showed a significantly decreased latency to lunge compared to CS_B_. Statistics revealed that this decrease can be explained by an effect due to the CS line (χ^2^test = 23.3, d.f = 1, *p* = 1e-06) and not the diet (χ^2^test = 1.5, d.f = 1, *p* = 0.2). **(B)** The number of meetings before the first lunge was different between CS lines [*F*_(1, 71)_ = 24.53, *p* = 5e-06), but was not affected by the diet [*F*_(1, 70)_ = 2.6, *p* = 0.11]. **(C)** CS_A_ lunged significantly more than CS_B_ [*F*_(1, 84_) = 12.51, *p* = 6.6e-04]. The diet did not significantly affect this parameter [*F*_(1, 83)_ = 0.4, *p* = 0.53]. **(D)** CS_A_ line gave higher number of lunges per aggressive meeting than CS_B_. Both CS lines [*F*_(1, 66)_ = 41.94, *p* = 1.5e-08] and diets [*F*_(1, 65)_ = 5.63, *p* = 0.02) affected the average number of lunges per aggressive meetings. **(E)** Fight outcomes were affected by the CS line (χ^2^test = 9.14, d.f = 1, *p* = 0.002), but not by the diet (χ^2^test = 1.54, d.f = 1, *p* = 0.21). **(F)** The latency to dominance was not different between CS line (χ^2^test = 0.7, d.f = 1, *p* = 0.4) and between diet (χ^2^test = 2.6, d.f = 1, *p* = 0.1). All the behavioral experiments were performed between ZT0 and ZT3. *P < 0.05, **P < 0.01, ***P < 0.001, ns = not significant.

**Figure 2:**
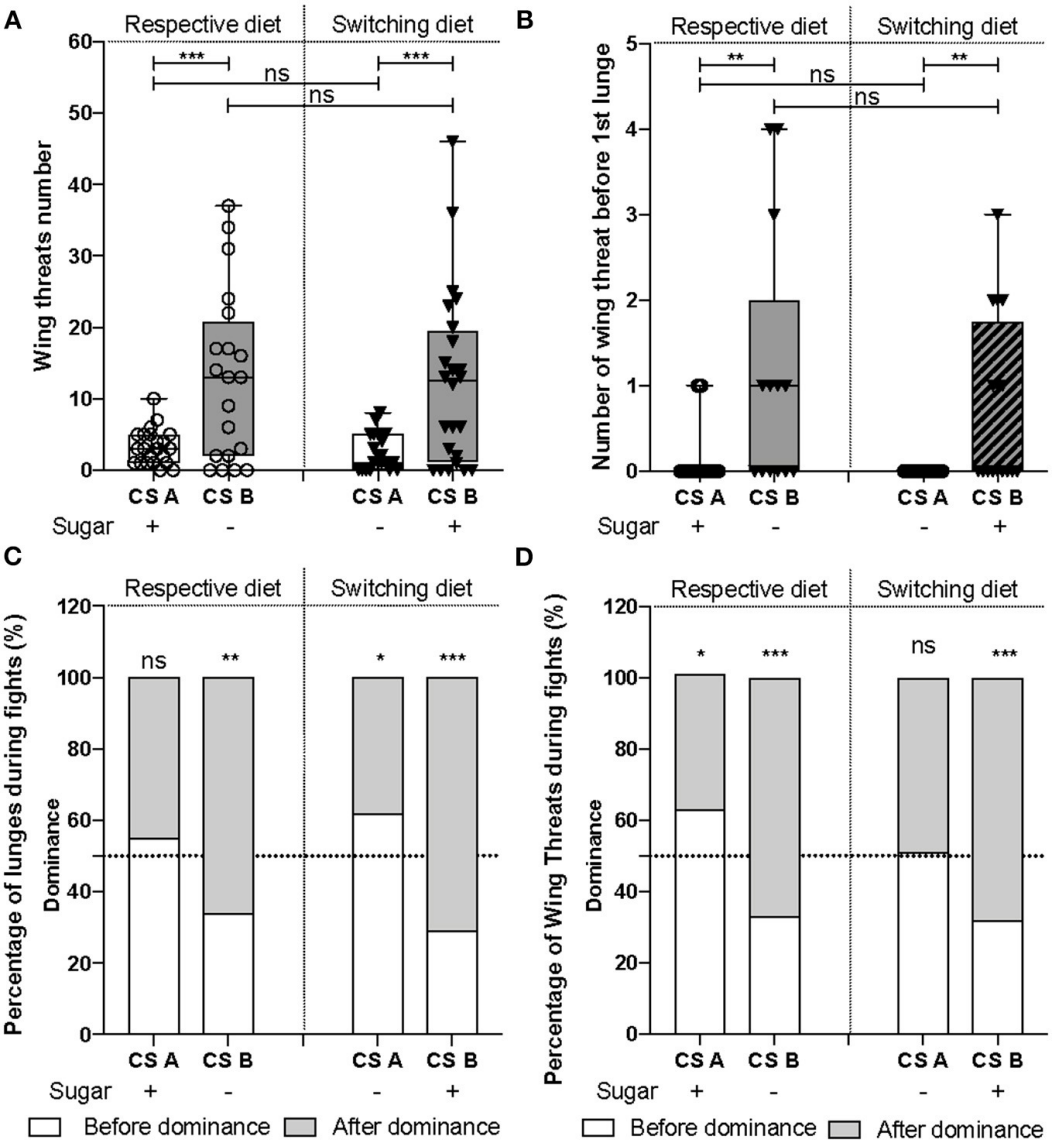
The two CS lines employed different fighting strategies. **(A)** The number of wing threats [*F*_(1, 85_) = 41.98, *p* = 6e-09] and **(B)** the number of wing threats before the first lunge [*F*_(1, 66)_ = 30.32, *p* = 6.9e-07] were significantly higher in CS_B_ line than CS_A_. However, diet does not influence **(A)** the display of wing threats [*F*_(1, 84)_ = 0.004, *p* = 0.94], nor **(B)** the number of wing threats before the first lunge [*F*_(1, 65)_ = 0.34, *p* = 0.56]. **(C)** CS_A_ lunged equally often before and after dominance was established (χ^2^test_CSAsugar+_ = 1, d.f = 1, *p* = 0.32; χ^2^test_CSAsugar−_ = 5.7, d.f = 1, *p* = 0.02), while CS_B_, preferentially lunged after dominance (χ^2^test_CSBsugar+_ = 17.64, d.f = 1, *p* = 0.0001; χ^2^test_CSBsugar−_ = 10.24, d.f = 1, *p* = 0.0014). **(D)** In CS_A_, wing threats were displayed throughout the fight (χ^2^test_CSAsugar+_ = 6.18, d.f = 1, *p* = 0.0129; χ^2^test_CSAsugar−_ = 0.04, d.f = 1, *p* = 0.84), while in CS_B_, they were observed mostly after dominance (χ^2^test_CSBsugar+_ = 12.96, d.f = 1, *p* = 0.0003; χ^2^test_CSBsugar−_ = 11.56, d.f = 1, *p* = 0.0007). *P < 0.05, **P < 0.01, ***P < 0.001, ns, not significant.

**Figure 3:**
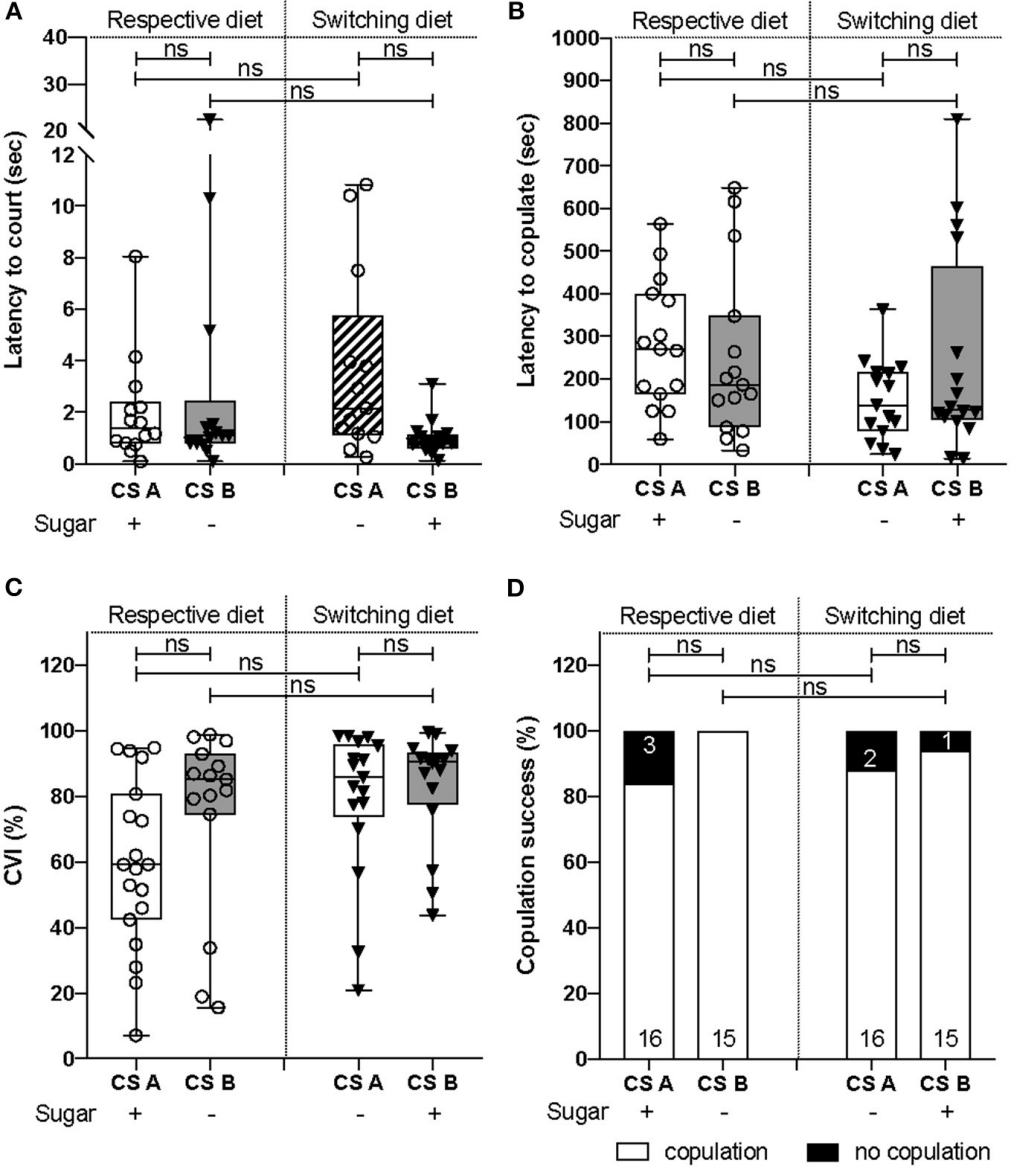
Courtship abilities did not differ between both CS lines. **(A)** There was no difference in the latency to court between CS lines (χ^2^test = 1.2, d.f = 1, *p* = 0.3) and diets (χ^2^test = 0.4, d.f = 1, *p* = 0.6). **(B)** No difference were observed across CS lines (χ^2^test = 1.7, d.f = 1, *p* = 0.2) and diets (χ^2^test = 2.5, d.f = 1, *p* = 0.1) for the latency to copulate. **(C)** CS lines [*F*_(1, 64)_ = 1.94, *p* = 0.18) and diet [*F*_(1, 64)_ = 3.93, *p* = 0.052] did not influence CVI. **(D)** The copulation success did not vary according to the CS lines (χ^2^test = 2.67, d.f = 1, *p* = 0.1) nor the diets (χ^2^test = 0.53, d.f = 1, *p* = 0.46).

**Figure 4:**
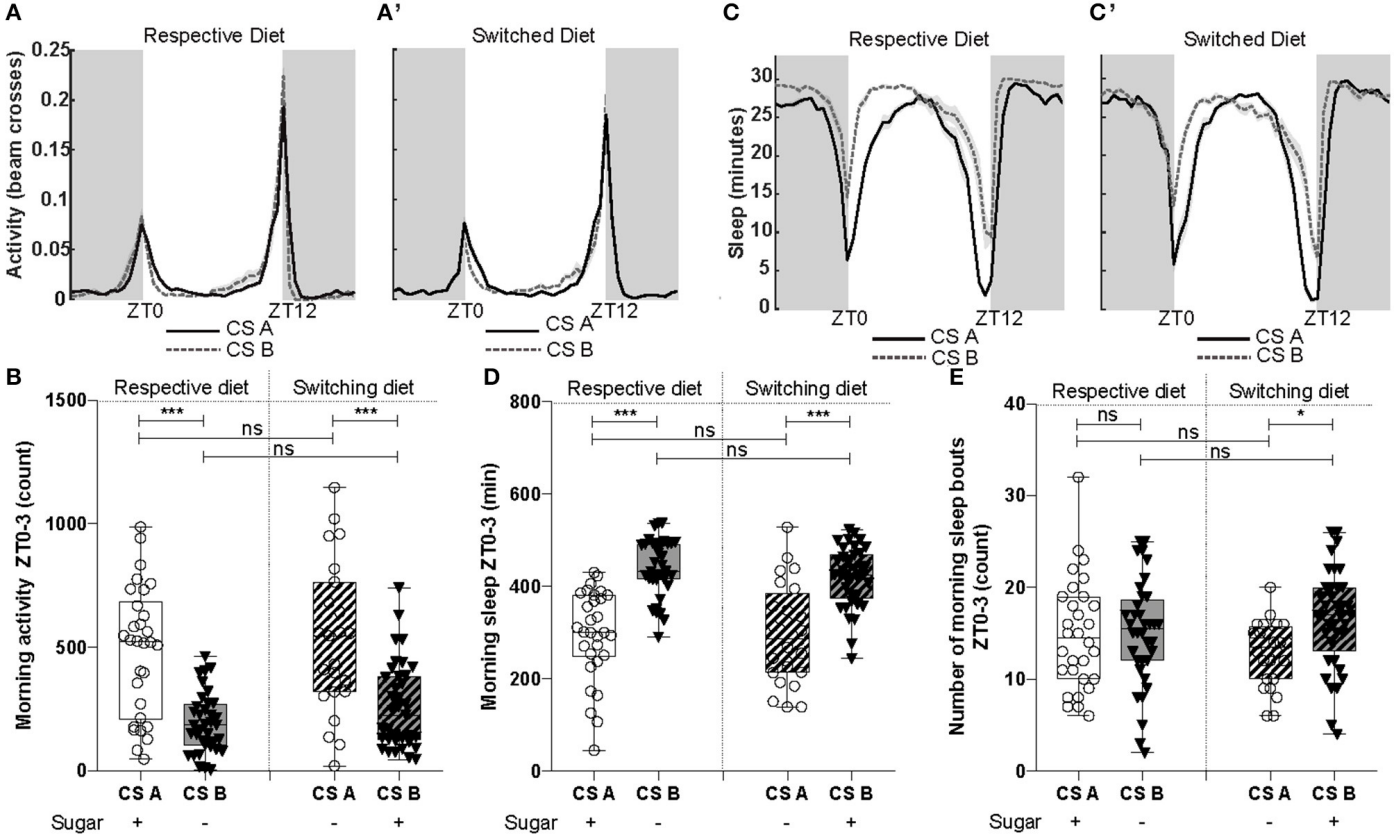
Cs line presented differences in their activity and sleep patterns. **(A)** Representation of activity patterns across day (white) and night (gray) for both lines when raised on their respective or **(A')** switching diet. **(B)** The CS_A_ line was significantly more active during the morning (ZT0-3) when behavioral experiments were done [*F*_(1, 124)_ = 55.76, *p* = 1.34e-11]. Diet did not affect morning activity [*F*_(1, 123)_ = 0.29, *p* = 0.58]. **(C)** Sleep patterns of CS lines when raised on their respective and **(C')** switching diets. **(D)** The line CS_B_ slept significantly more during the first 3 h of the morning [*F*_(1, 125)_ = 73,88, *p* = 3.16e-14]. Diet did not significantly affect morning sleep [*F*_(1, 124)_ = 0.42, *p* = 0.51]. **(E)** Number of morning sleep bouts (ZT0-3) were not significantly different between CS lines [*F*_(1, 125)_ = 3.72, *p* = 0.056] and diets [*F*_(1, 124)_ = 3.51, *p* = 0.064], even though a statistical difference was detected by the post-test between CS lines when raised on switching diet. *P < 0.05, ***P < 0.001, ns, not significant.

GraphPad Prism 8 was used to assess the normality of the distribution with the Shapiro-Wilk test and to identify outlier values with a Grubb's test (alpha = 0.05). R software was used to assess the effects of the factors “CS line” and “Diet” as well as the possible interaction between them, by using distinct statistical methods according to the nature of the behavioral response tested:

- For all latencies (Figures 1A,F, **3A,B** and Supplementary Figure 2): survival analysis of Kaplan-Meier (survdiff) with a χ^2^test.
- For binomial distribution (Figures 1E, **3D**): logistic regression (lm) model with a χ^2^test to assess the effect of both factors, followed by a Tukey post-test.
- For percentages (**Figure 3C**): logistic regression (lm) with an ANOVA to assess the effect of both factors, followed by a Tukey post-test.
- All numerical measurements (Figures 1B–D, 2A,B, **4B,D,E** and Supplementary Figures 4, 5): Generalized linear model (glm) with Quasi-Poisson error distribution was used to overcome the non-Gaussian distribution of the data. The significance of the effect due to the CS line, diet or the interaction, was assessed using an ANOVA followed by a Tukey post-test.
- For data distribution (Figures 2C,D and Supplementary Figure 3): χ^2^test to compare to the expected value of 50%. The tests were done with GraphPad online software.

Differences were considered statistically significant at *p* < 0.05. Data are presented as boxplots including all data points. The lower and upper edges of each box correspond to the 25 and 75% quantiles, respectively. Percentages are presented as stacked bars. All the statistics can be found in Supplementary Table 3.

## Results

### Dietary Sugar Content Leads to Different Aggression Levels

To assess whether dietary regimes might have driven adaptation of aggressive behavior, we collected two CS lines that serve as reference lines in two laboratories: CS_A_ and _B_ lines, that were raised for ~10 years on either sugar-containing or sugar-free diets, respectively. We set up male-male aggression assays and scored behavioral parameters. When comparing the latency to lunge, we observed that CS_A_ males started fighting significantly sooner (Figure 1A, left panel “respective diet”), with a tendency to meet fewer times before the first lunge (Figure 1B, left panel), suggesting that CS_A_ males have a higher motivation to fight compared to CS_B_. As the latency to lunge was significantly increased in the CS_B_ line, we assessed the aggressiveness level of both lines by scoring the number of lunges displayed within 10 min since the first lunge (as opposed to quantifying it for a fixed amount of time after the first meeting). CS_B_ males showed a significant reduction of the total number of lunges (Figure 1C, left panel), as well as of the average number of lunges per aggressive meetings (Figure 1D, left panel), showing that aggressiveness level was reduced in the CS_B_ line compared to CS_A_. In the same way, CS_B_ fought in only 65% of assays (assays with at least one lunge) compared to 100% for CS_A_ (Figure 1E, left panel). However, no difference was observed in the latency to dominance Figure 1E, left panel). Together, these results demonstrate that the two CS populations raised on their respective diets for almost 10 years differed by their motivation to start fighting, their aggressiveness level, and fight outcomes.

### Aggression Level Is Negatively Impacted by Long-Term Sugar-Restriction

Next, we asked whether switching diets would affect aggressive behavior of both CS lines. Would raising CS_B_ on a sugar-containing diet rescue the diminution of their aggressiveness level? On the contrary, would depriving CS_A_ from sugar negatively impact males' aggression level? To address these questions, we raised the CS_A_ line on sugar-free diet and CS_B_ line on sugar-containing diet for 3 months and performed male-male aggression assays – an experimental condition called “switching diet.” Behavioral experiments on respective and switching diets were done in parallel to compare aggressive patterns of CS_A_ and CS_B_ B when raised on both diets. No significant differences were found (Figure 1, entire panel), except for the average number of lunges per aggressive meetings (Figure 1D). However, when comparing both CS_A_ and CS_B_ raised on switching diets, we still observed that the CS_B_ line showed a reduction in their motivation to fight, exhibited fewer lunges per aggressive encounter, and fought less often (Figures 1A–F, right panels), recapitulating the results observed when raised on their respective diets. This “switching diet” experimental condition indicated that depriving CS_A_ flies from sugar did not reduce their aggressiveness. In agreement with this, raising CS_B_ flies on a diet containing sugar did not either enhance aggressiveness. These results demonstrate that the motivation to fight, aggressiveness, and fight outcomes could not be restored by switching diets for 3 months, rather they suggest that they potentially result from a longer-term influence of diet, and a behavioral adaptation to an environmental condition.

### CS Lines Have Developed Distinct Fighting Strategies

Employing high-intensity lunges throughout the fight remains an efficient fighting strategy used by males to establish a stable dominance relationship. However, as CS_B_ males formed dominance relationships while using fewer lunges than CS_A_ during fights (Figure 1), we asked whether they have developed an alternative fighting strategy to attain and maintain this social consensus between competitors. For this, we scored the number of wing threats and found that CS_B_ displayed significantly more of these visual threats than did CS_A_ (Figure 2A). Moreover, CS_B_ displayed more wing threats before the first lunge (Figure 2B). Indeed, in 54% (7/13) and 47% (8/17) of assays, CS_B_ males displayed wing threats as the first aggressive demonstration when raised on their respective and switching diets, respectively, while these observations dropped to 13% (3/22) and 0.05% (1/18) for CS_A_. However, the latency to display the first wing threat was not different (Supplementary Figure 2). This shows that sugar-free raised-males may have developed an alternative fighting strategy in which dominance could be formed and maintained by using fewer lunges but more threats. To further explore this hypothesis, we compared the percentages of lunges (Figure 2C) and wing threats (Figure 2D) given before and after dominance, to the random value of 50%. A value near 0% implies that most of the lunges were given before dominance, a value near 50% implies that flies lunged before and after dominance equally, and a value near 100% implies that most lunges occurred after dominance. We observed that CS_A_ raised on their respective and switching diets, respectively, exhibited 55 and 62% of the lunges (Figure 2C) and 63 and 51% of the wing threats (Figure 2D) before establishment of dominance, showing that aggressive patterns are almost equally distributed throughout the fight. On the contrary, CS_B_ males exhibited only 34 and 29% of the lunges (Figure 2C) and 33 and 32% of the wing threats (Figure 2D) before dominance, showing that they are preferentially distributed after dominance was established. As the majority of lunges and wing threats were displayed by the winners (Supplementary Figure 3), they are likely used by dominants to establish and maintain dominance relationships. Altogether, these results demonstrate that males from the two CS lines employ distinct fighting strategies to establish and maintain dominance: CS_A_ started fights with lunges, while CS_B_ with either wing threats or lunges. Also, CS_A_ favored the use of lunges to establish and maintain dominance, while CS_B_ preferentially used both behavioral patterns to maintain it.

### Courtship Performances and Reproductive Capacities Are Not Affected by Diet

We next investigated whether males would also employ distinct reproductive strategies in a male-to-female courtship context. We therefore set up courtship assays involving one CS_A_ or CS_B_ male with a female from the same line, and scored courtship behavior. We observed that the latencies to court and to copulate were not statistically different between lines or when lines were raised on either diet (Figures 3A,B). In the same way, male courtship performances did not differ significantly between lines and diets (Figure 3C). Finally, the copulation success rate was not statistically different between CS lines (Figure 3D). These results demonstrate that there were no differences in courtship performances and reproductive abilities between both CS lines.

### CS Lines Showed Differences in Their Activity and Sleep Patterns

A reduction of aggression could come from a reduction of locomotor activity. Therefore, we performed activity and sleep experiments with males of both lines using either their respective and switched diet. When restricting the analysis to the first 3 h of the day (Zeitgeber time 0-3, ZT0-ZT03) to match the time when aggression experiments were performed, we observed differences in both parameters (Figure 4). Males of the CS_B_ line exhibited lower levels of activity in this specific time window regardless of the diet (Figures 4A,B). Consistently with this observation, we noticed an increase in total sleep for the CS_B_ line compared to CS_A_, in both diets (Figures 4C,D). However, for the total number of sleep bouts (5-min period of inactivity), we only noticed a mild increase for CS_B_ in their switched diet (Figure 4E). When analyzing the same parameters during the day (ZT0-ZT12) or night (ZT12-ZT24) phases, CS_B_ only showed significant decreases in activity relative to CS_A_ when analyzed in their respective diet (Supplementary Figure 4). In the case of sleep, only minutes of day sleep was increased for CS_B_ in both diets but not sleep bouts (Supplementary Figures 5A,B). Night sleep showed no differences between lines (Supplementary Figures 5C,D).

## Discussion

Dietary regimes play crucial roles in the life history of animals and can affect their behaviors in many ways (Tremmel and Müller, 2013; Han and Dingemanse, 2015). Animals living in changing environments must develop adaptive behavioral responses to withstand dietary challenging situations (Partridge and Brand, 2005; Adler et al., 2013). In Drosophila, manifestation of aggressive behavior is subjected to modifications by environmental and social conditions (Svetec and Ferveur, 2005; Bath et al., 2018; Kilgour et al., 2019). To further follow these studies, we investigated the behavioral plasticity of Drosophila aggressive behaviors in response to two distinct dietary regimes. The long-term diet-related consequences are a modification of the fighting motivational state, aggressiveness level, and fighting strategy employed to reach dominance.

In addition to its essential function of nutrition, diets modulate behavioral expression and ultimately control social interactions, including aggression (Wallner, 2009). Here, we revealed that male flies raised on sugar-containing diet are overall more aggressive than those raised on a sugar-free diet. We also showed that visual threats are another key component of Drosophila fights. Indeed, sugar-free raised-flies showed fewer lunges but more wing threats, which significantly impacted the fight dynamics and modified the fighting strategy to reach and maintain dominance. Based on our results, we propose that, in addition to lunges, wing threats should be considered as an informative behavioral pattern when studying fighting strategies and establishment of dominance relationships in *Drosophila* males. Our results demonstrate that the reduction in lunging behavior and fighting motivational state can't be rescued by switching diets, suggesting that the diet-induced males' aggression phenotype observed results from a long-term behavioral adaptation to diet. However, investigating aggression in a female-female context would provide additional information about how dietary regimes influence fighting strategies in general, and would reinforce our current hypothesis.

Raising flies on two different diets does not interfere with males' courtship performance, nor with their reproduction capacities between males and females from the same CS line. Performing courtship experiments with reciprocal females, however, could affect these parameters. In the same way, performing competitive courtship assays could reveal whether a preference for a non-random mating has emerged in these lines after years of potential experimental evolution, which has been observed when investigating the emergence of behavioral isolation (Belkina et al., 2018).

CS males raised on sugar-free diet also show a reduction in their locomotor activity and an increase in sleep patterns, particularly during the daytime. This could account for some of the described aggression phenotypes, like latency to the first lunge, but not for all. In the latter case, we could expect increases in both latencies to lunge and to dominance, and a reduction in all aggressive patterns, including wing threats. Also, we would expect differences in their latency to court and/or copulate with females, which was not the case. Therefore, differences in activity and/or sleep levels do not explain what we consider the most salient aspects of the behavioral differences: the frequency of wing threat displays leading to modification of fight's dynamics and the development of alternative fighting strategy to reach dominance.

Our findings support previous observations that animals fed with low sucrose diet are less aggressive than those fed with high level of sucrose (Grover et al., 2007; Haagensen et al., 2014; Meichtry et al., 2020). From a physiological point of view, as the production of ATP from the conversion of carbohydrates is a key source of energy for insects, exposure to low sugar or sugar-free diets might have forced animals to develop less energy-consuming fighting strategies, while staying competitive toward others. However, sugar-deprived diets may have additional consequences. Indeed, insects use cuticular hydrocarbons (CHC), acting as pheromones, to drive social behaviors (Yew and Chung, 2017). For example, changes in the amount of 11-cis-vaccenyl acetate (cVa) pheromone modulate male courtship behavior (Ejima, 2015) and aggression by altering the number of lunges (Fernandez et al., 2010; Wang et al., 2011). As diet (Fedina et al., 2012) and circadian rhythm (Krupp et al., 2008) influence the CHC profile of flies, it is possible that CS lines present differences in the amount of some CHC, leading to changes in the expression of lunge behavior. Another explanation would be that diet affects anterior inferior protocerebrum (AIP) neuronal activity, recently described to specifically control threat displays without affecting other types of agonistic behavior (Duistermars et al., 2018). To further follow this work on behavioral adaptation to diets, it would be interesting to perform whole brain RNAi sequencing on these CS lines. This would allow to identify whether genes already known to control social behaviors are differentially expressed between these lines in response to distinct dietary regimes.

In sum, our results show that fruit flies raised for years under different dietary conditions have adapted their aggressive behaviors and developed two distinct fighting strategies: one favoring physical attacks, while the other one employing both physical attacks and visual threats. This shows the long-term influence of diet-based environmental pressure on aggression and adaptation of animals' fighting strategies.

## Data Availability Statement

The raw data supporting the conclusions of this article will be made available by the authors upon request.

## Ethics Statement

Despite the widespread use of invertebrates in research, only few ethical guidelines exist and applied to crustaceans and cephalopods. However, using Drosophila as a model for behavioral research also involve experimental design planification and the use of anesthetic methods for reducing animals' pain and suffering during and after experiments (Drinkwater et al., 2019).

## Author Contributions

JL performed preliminary experiments on CS_A_ and _B_ lines. GT and MF performed and analyzed activity and sleep patterns, and edited the paper. JG and ST wrote the scripts for statistical analysis in R software. ST designed the study, performed, analyzed and scored all behavioral experiments, performed statistical analysis, and wrote the paper. All the authors gave final approval for submission.

## Conflict of Interest

The authors declare that the research was conducted in the absence of any commercial or financial relationships that could be construed as a potential conflict of interest.
