# Supplementary figures and images for: Long-Term Dietary Restriction Leads to Development of Alternative Fighting Strategies
Source: Front Behav Neurosci. 2021 Jan 14;14:599676. doi: 10.3389/fnbeh.2020.599676 (PMC7840567; doi:10.3389/fnbeh.2020.599676)

## Figure Supp 1

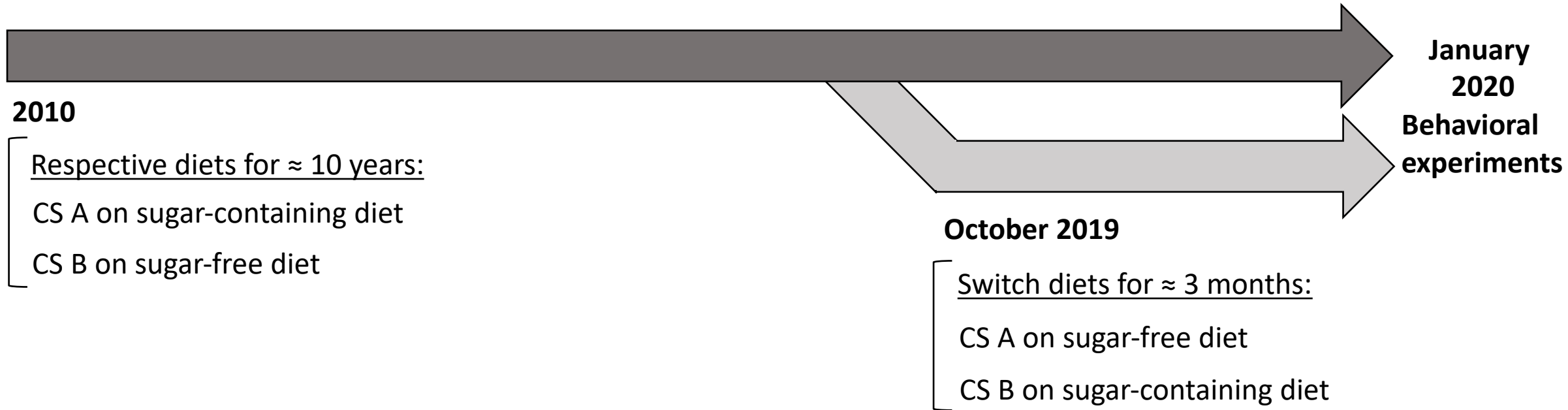

Supplement: Supplementary file 1 [file Data_Sheet_1.PDF]

**Figure Supp 2**

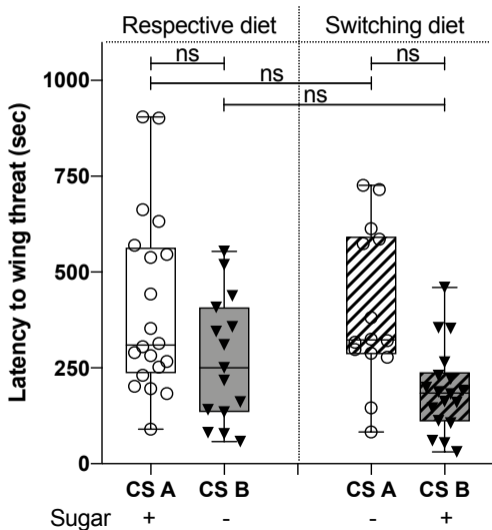

Supplement: Supplementary file 2 [file Data_Sheet_2.PDF]

Figure Supp 3

A

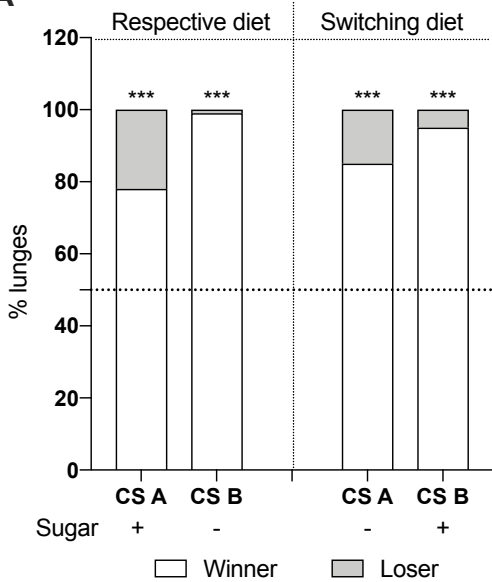

B

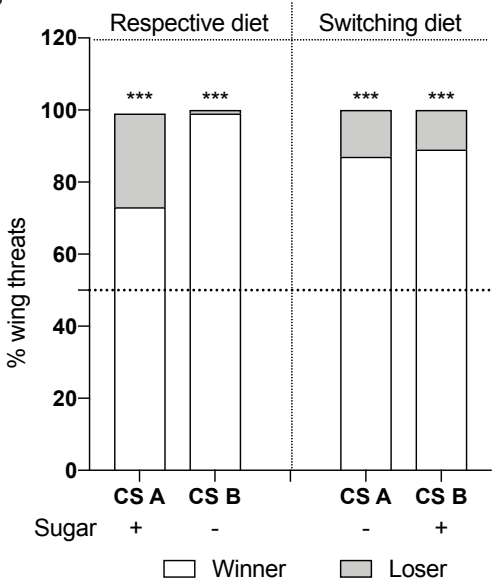

Supplement: Supplementary file 3 [file Data_Sheet_3.PDF]

**Figure Supp 4**

**A**

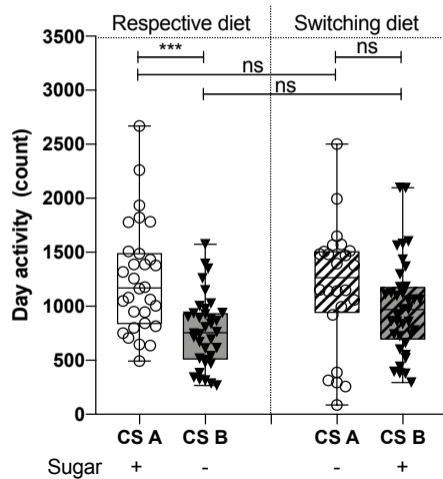

**B**

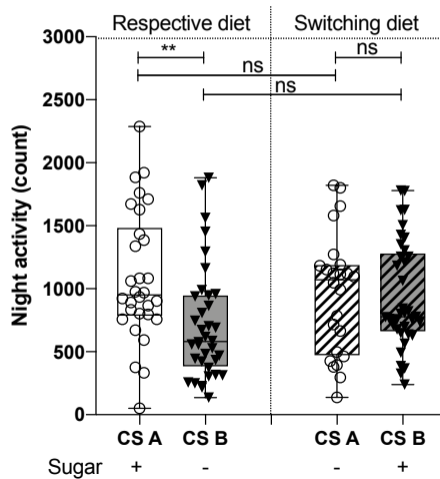

**C**

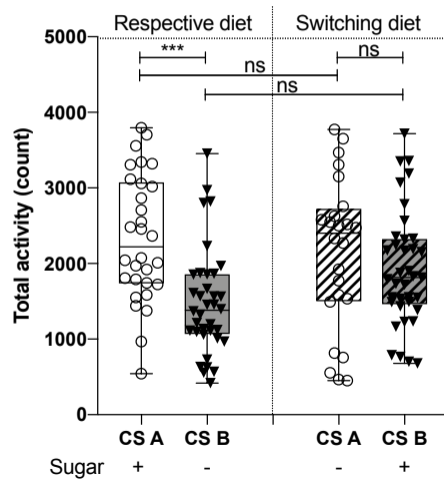

Supplement: Supplementary file 4 [file Data_Sheet_4.PDF]

Figure Supp 5

A

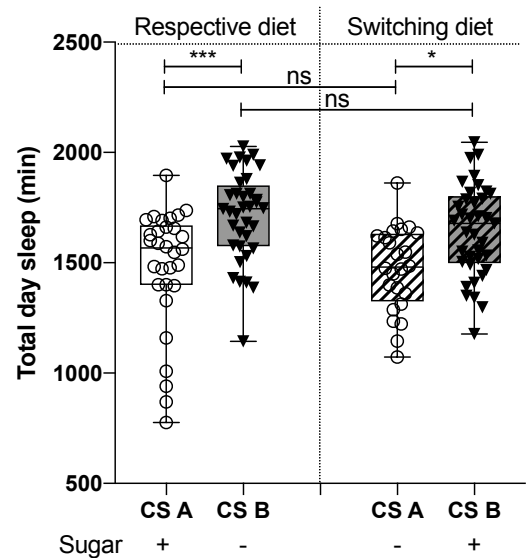

B

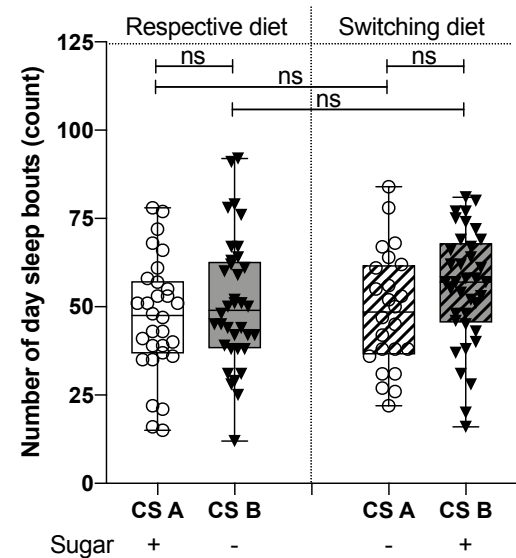

C

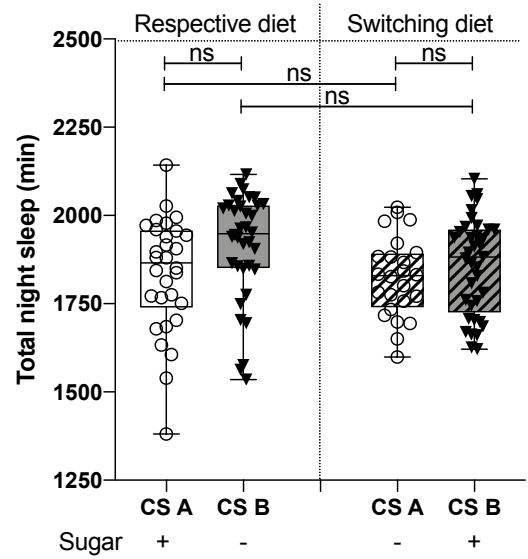

D

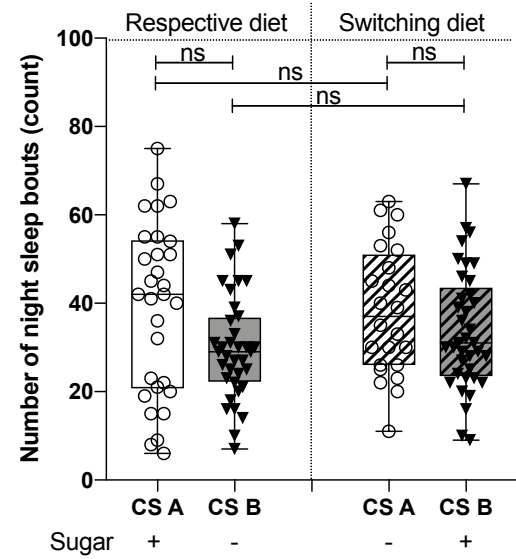

Supplement: Supplementary file 5 [file Data_Sheet_5.PDF]
