# Supplementary material for: Long-Term Dietary Restriction Leads to Development of Alternative Fighting Strategies
Source: Front Behav Neurosci. 2021 Jan 14;14:599676. doi: 10.3389/fnbeh.2020.599676 (PMC7840567; doi:10.3389/fnbeh.2020.599676)
Supplement: Supplementary file 6 [file Data_Sheet_6.PDF]

Table Supp1

	Diet containig (%)		
	sugar	yeast	floor/cornmeal
Before October 2019			
CS A	121	27,5	52
CS B	0	70	70
Since October 2019			
CS A	40	28	74
CS B	0	70	70
