# Supplementary material for: Long-Term Dietary Restriction Leads to Development of Alternative Fighting Strategies
Source: Front Behav Neurosci. 2021 Jan 14;14:599676. doi: 10.3389/fnbeh.2020.599676 (PMC7840567; doi:10.3389/fnbeh.2020.599676)
Supplement: Supplementary file 7 [file Data_Sheet_7.PDF]

Table Supp 2: Scoring aggressive behavior

Behavioral parameter	Definition	Related to figure :
Meeting	Physical interaction between both flies for at least two secondes	1
Aggressive meeting	Meeting involving at least one lunge by one fly	1
Lunge	Physical attack: one fly raises on its back legs and snaps down to the other fly	1
Wing threat	Visual threat: one fly raises both wings at 45° towards its opponent	2
Latency to lunge	Time in secondes between the first meeting and the first lunge	1
Dominance	Time when the putative loser retreats from the food cup three times after having received lunges from the other	1
Latency to wing threat	Time in secondes between the first meeting and the first wing threat	2
Latency to dominance	Time in secondes between the first meeting and the establishment of dominance	1
Fight outcome	No fight: 0 lunges were observed during the 15 min of observation	1
	Draw: lunges were observed but were not sufficient to induce dominance or retaliation was observed betwenn opponents	
	Dominance: dominance has been established between competitors during the 15 min of observation	
Percentage of lunges during fights (%)	Percentage of total number of lunges scored before and after establishment of dominance	2
Percentage of wing threats during fights (%)	Percentage of total number of wing threats scored before and after establishment of dominance	2
