# Supplementary material for: Long-Term Dietary Restriction Leads to Development of Alternative Fighting Strategies
Source: Front Behav Neurosci. 2021 Jan 14;14:599676. doi: 10.3389/fnbeh.2020.599676 (PMC7840567; doi:10.3389/fnbeh.2020.599676)
Supplement: Supplementary file 8 [file Data_Sheet_8.PDF]

FIGURE 1	Diet	CS line	n	Mean	Std. Dev.	S.E.M.	Gaussian distribution	Test					Post-test		
**A. Latency to lunge**								**survival analysis of Kaplan-Meier**	**factor**	**Chisq**	**p value**	**summary**			
	Respective	CS A	21	35.52	50.29	10.97	No		CS line	23.3	0.000001	***			
		CS B	13	166.9	155.5	43.13	No		Diet	1.5	0.2	ns			
	Switch	CS A	17	46.59	60.09	14.57	No								
		CS B	17	170.5	194.7	47.21	No								
**B. Number of meeting before 1st lunge**								**Generalized linear model (glm)**	**factor**	**F**	**p value**	**summary**	**Tukey post-test**	**p value**	**summary**
	Respective	CS A	21	1.524	0.6016	0.1313	No		CS line	24.53	0.000005	***	CS A resp Vs CS B resp	0.067	ns
		CS B	17	3.706	3.619	0.8777	No		Diet	2.6	0.111	ns	CS A switch Vs CS B switch	0.0003	***
	Switch	CS A	17	1.882	1.054	0.2556	No		Interaction CS line * Diet	2.5	0.121	ns	CS A resp Vs CS A switch	0.957	ns
		CS B	18	6.444	6.644	1.566	No						CS B resp Vs CS B switch	0.126	ns
**C. Number of lunges**								**Generalized linear model (glm)**	**factor**	**F**	**p value**	**summary**	**Tukey post-test**	**p value**	**summary**
	Respective	CS A	21	32.24	20.3	4.43	Yes		CS line	12.51	0.00067	***	CS A resp Vs CS B resp	0.015	*
		CS B	20	13.95	12.93	2.891	No		Diet	0.4	0.527	ns	CS A switch Vs CS B switch	0.168	ns
	Switch	CS A	21	32.05	25.76	5.622	Yes		Interaction CS line * Diet	0.68	0.412	ns	CS A resp Vs CS A switch	1	ns
		CS B	24	19.21	20.03	4.088	No						CS B resp Vs CS B switch	0.721	ns
**D. Average lunges per aggressive meetings**								**Generalized linear model (glm)**	**factor**	**F**	**p value**	**summary**	**Tukey post-test**	**p value**	**summary**
	Respective	CS A	22	3.273	1.51	0.3219	Yes		CS line	41.94	1.53E-08	***	CS A resp Vs CS B resp	0.002	**
		CS B	12	1.608	0.466	0.1345	Yes		Diet	5.63	0.021	*	CS A switch Vs CS B switch	<.0001	***
	Switch	CS A	17	4.882	2.938	0.7125	No		Interaction CS line * Diet	2.83	0.097	ns	CS A resp Vs CS A switch	0.024	*
		CS B	17	1.741	0.568	0.1377	Yes						CS B resp Vs CS B switch	0.989	ns
**E. Fight Outcome (%)**			**Fight (%)**	**No fight (%)**				**logistic regression (lm)**	**factor**	**Dev. Resid.**	**p value**	**summary**	**Tukey post-test**	**p value**	**summary**
	Respective	CS A	22	0					CS line	9.145	0.003	**	CS A resp Vs CS B resp	0.006	**
		CS B	13	7					Diet	1.542	0.214	ns	CS A switch Vs CS B switch	0.602	ns
	Switch	CS A	18	3					Interaction CS line * Diet	3.165	0.075	ns	CS A resp Vs CS A switch	0.241	ns
		CS B	17	7									CS B resp Vs CS B switch	0.976	ns
**F. Latency to dominance**								**survival analysis of Kaplan-Meier**	**factor**	**Chisq**	**p value**	**summary**			
	Respective	CS A	18	301.9	184.7	43.54	Yes		CS line	0.7	0.4	ns			
		CS B	13	311.2	164.7	45.68	Yes		Diet	2.6	0.1	ns			
	Switch	CS A	11	284.7	169.5	51.12	Yes								
		CS B	15	319.6	242.0	62.49	No								

FIGURE 2	Diet	CS line	n	Mean	Std. Dev.	S.E.M.	Gaussian distribution	Test					Post-test		
**A. Wing threats number**			22	3,273	2,492	0,5312	Yes	**Generalized linear model (glm)**	**factor**	**F**	**p value**	**summary**	**Tukey post-test**	**p value**	**summary**
	Respective	CS A	20	13	11,85	2,65	No		CS line	41.982	6.18E-09	***	CS A resp Vs CS B resp	0.0002	***
		CS B	21	2,429	2,521	0,5502	No		Diet	0.004	0.948	ns	CS A switch Vs CS B switch	<.0001	***
	Switch	CS A	24	12,38	12,18	2,486	No		Interaction CS line * Diet	0.434	0.512	ns	CS A resp Vs CS A switch	0.924	ns
		CS B											CS B resp Vs CS B switch	0.996	ns
**B. Number of wing threats before 1st lunge**								**Generalized linear model (glm)**	**factor**	**F**	**p value**	**summary**	**Tukey post-test**	**p value**	**summary**
	Respective	CS A	22	0,1364	0,3513	0,07489	No		CS line	30.324	6.926E-07	***	CS A resp Vs CS B resp	0.005	**
		CS B	13	1,154	1,519	0,4213	No		Diet	0.344	0.559	ns	CS A switch Vs CS B switch	0.003	**
	Switch	CS A	17	0	0	0	ND		Interaction CS line * Diet	4.413	0.039	*	CS A resp Vs CS A switch	0.300	ns
		CS B	16	0,75	1	0,25	No						CS B resp Vs CS B switch	0.686	ns
**C. Percentage of lunges during fights (%)**			**Before dominance**	**After dominance**				**Chi2 test**	To 50% expected value	**p value**	**summary**				
	Respective	CS A	55	45					1.0	0.317	ns				
		CS B	34	66					10.24	0.002	**				
	Switch	CS A	62	38					5.760	0.017	*				
		CS B	29	71					17.640	<.0001	***				
**D. Percentage of wing threats during fights (%)**			**Before dominance**	**After dominance**				**Chi2 test**		**p value**	**summary**				
	Respective	CS A	63	38					6.188	0.013	*				
		CS B	33	67					11.560	0.0007	***				
	Switch	CS A	51	49					0.040	0.841	ns				
		CS B	32	68					12.960	0.0003	***				

FIGURE 3	Diet	CS line	n	Mean	Std. Dev.	S.E.M.	Gaussian distribution	Test					Post-test		
**A. Latency to court**								**survival analysis of Kaplan-Meier**	**factor**	**Chisq**	**p value**	**summary**			
	Respective	CS A	18	2,639	2,784	0,6562	No		CS line	1.2	0.3	ns			
		CS B	14	3,441	6,074	1,623	No		Diet	0.4	0.6	ns			
	Switch	CS A	17	3,002	3,382	0,8202	No								
		CS B	16	0,9906	0,667	0,1667	No								
**B. Latency to copulate**								**survival analysis of Kaplan-Meier**	**factor**	**Chisq**	**p value**	**summary**			
	Respective	CS A	15	282,9	147,5	38,08	Yes		CS line	1.7	0.2	ns			
		CS B	15	249,8	199,3	51,47	No		Diet	2.5	0.1	ns			
	Switch	CS A	15	151,8	93,85	24,23	Yes								
		CS B	16	247,4	239,9	59,98	No								
**C. CVI (%)**								**logistic regression (lm)**	**factor**	**F**	**p value**	**summary**	**Tukey post-test**	**p value**	**summary**
	Respective	CS A	19	59,27	25,59	5,87	Yes		CS line	1.842	0.179	ns	CS A resp Vs CS B resp	0.295	ns
		CS B	15	74,57	27,93	7,212	No		Diet	1.946	0.168	ns	CS A switch Vs CS B switch	1.000	ns
	Switch	CS A	17	79,08	22,76	5,519	No		Interaction CS line * Diet	3.933	0.052	ns	CS A resp Vs CS A switch	0.092	ns
		CS B	16	82,92	17,24	4,311	No						CS B resp Vs CS B switch	0.961	ns
**D. Copulation success (%)**			**Copulation**	**No copulation**				**logistic regression (lm)**	**factor**	**Dev. Resid.**	**p value**	**summary**	**Tukey post-test**	**p value**	**summary**
	Respective	CS A	84	16					CS line	2.676	0.102	ns	CS A resp Vs CS B resp	0.233	ns
		CS B	100	0					Diet	0.535	0.464	ns	CS A switch Vs CS B switch	0.929	ns
	Switch	CS A	88	12					Interaction CS line * Diet	0.881	0.348	ns	CS A resp Vs CS A switch	0.985	ns
		CS B	94	6									CS B resp Vs CS B switch	0.731	ns

FIGURE 4	Diet	CS line	n	Mean	Std. Dev.	S.E.M.	Gaussian distribution	Test					Post-test		
**B. Morning activity ZT0-3**								**Generalized linear model (glm)**	**factor**	**F**	**p value**	**summary**	**Tukey post-test**	**p value**	**summary**
	Respective	CS A	30	484,7	261,5	47,74	Yes		CS line	55.764	1.348E-11	***	CS A resp Vs CS B resp	<.0001	***
		CS B	36	193	120	19,99	Yes		Diet	0.299	0.585	ns	CS A switch Vs CS B switch	<.0001	***
	Switch	CS A	23	531,4	306,4	63,9	Yes		Interaction CS line * Diet	3.193	0.076	ns	CS A resp Vs CS A switch	0.906	ns
		CS B	37	260,6	174,9	28,76	No						CS B resp Vs CS B switch	0.303	ns
**D. Morning sleep ZT0-3**								**Generalized linear model (glm)**	**factor**	**F**	**p value**	**summary**	**Tukey post-test**	**p value**	**summary**
	Respective	CS A	30	295,7	97,85	17,87	Yes		CS line	73.878	3.157E-14	***	CS A resp Vs CS B resp	<.0001	***
		CS B	36	438,6	62,65	10,44	Yes		Diet	0.426	0.515	ns	CS A switch Vs CS B switch	<.0001	***
	Switch	CS A	24	296,2	109	22,25	Yes		Interaction CS line * Diet	0.190	0.664	ns	CS A resp Vs CS A switch	1.000	ns
		CS B	37	421,3	65,66	10,79	Yes						CS B resp Vs CS B switch	0.862	ns
**E. Number of morning sleep bouts ZT0-3**								**Generalized linear model (glm)**	**factor**	**F**	**p value**	**summary**	**Tukey post-test**	**p value**	**summary**
	Respective	CS A	30	14,87	6,021	1,099	Yes		CS line	3.720	0.056	ns	CS A resp Vs CS B resp	0.999	ns
		CS B	36	15	5,782	0,9636	Yes		Diet	3.506	0.063	ns	CS A switch Vs CS B switch	0.030	*
	Switch	CS A	24	12,83	3,559	0,7265	Yes		Interaction CS line * Diet	0.124	0.725	ns	CS A resp Vs CS A switch	0.467	ns
		CS B	37	16,59	5,377	0,884	Yes						CS B resp Vs CS B switch	0.599	ns

FIGURE SUPP 1	Diet	CS line	n	Mean	Std. Dev.	S.E.M.	Gaussian distribution	Test				
Latency to wing threat								survival analysis of Kaplan-Meier	factor	Chisq	p value	summary
	Respective	CS A	20	408,2	233,6	52,23	No		CS line	1.8	0.2	ns
		CS B	15	270,1	163,1	42,1	Yes		Diet	2.6	0.1	ns
	Switch	CS A	14	403,6	203,3	54,34	Yes					
		CS B	18	192,9	111,8	26,35	Yes					

FIGURE SUPP 2	Diet	CS line			Test			
A. % lunges			Winner (%)	Loser (%)	Chi2 test	To 50% expected value	p value	summary
	Respective	CS A	78	22		31.360	0.0001	***
		CS B	99	1		96,04	0,0001	***
	Switch	CS A	85	15		49.000	0.0001	***
		CS B	95	5		81.000	0.0001	***
B. % wing threats			Winner (%)	Loser (%)	Chi2 test	To 50% expected value	p value	summary
	Respective	CS A	73	27		21.160	0.0001	***
		CS B	99	1		96,04	0,0001	***
	Switch	CS A	87	13		54.760	0.0001	***
		CS B	89	11		60.840	0.0001	***

FIGURE SUPP 3	Diet	CS line	n	Mean	Std. Dev.	S.E.M.	Gaussian distribution	Test					Post-test		
A. Day activity								Generalized linear model (glm)	factor	F	p value	summary	Tukey post-test	p value	summary
	Respective	CS A	30	1251	504,3	92,06	Yes		CS line	17.627	5.11E-05	***	CS A resp Vs CS B resp	0.0001	***
		CS B	36	768,9	326,2	54,36	Yes		Diet	3.701	0.057	ns	CS A switch Vs CS B switch	0.375	ns
	Switch	CS A	24	1183	583,3	119,1	Yes		Interaction CS line * Diet	1.501	0.223	ns	CS A resp Vs CS A switch	0.958	ns
		CS B	37	986,1	439,6	72,26	Yes						CS B resp Vs CS B switch	0.116	ns
B. Night activity								Generalized linear model (glm)	factor	F	p value	summary	Tukey post-test	p value	summary
	Respective	CS A	30	1089	516,4	94,29	Yes		CS line	5.868	0.017	*	CS A resp Vs CS B resp	0.006	**
		CS B	36	709,5	447	74,49	No		Diet	5.221	0.024	*	CS A switch Vs CS B switch	0.999	ns
	Switch	CS A	24	954,3	482,5	98,48	Yes		Interaction CS line * Diet	0.640	0.425	ns	CS A resp Vs CS A switch	0.749	ns
		CS B	37	938,2	420	69,04	No						CS B resp Vs CS B switch	0.121	ns
C. Total activity								Generalized linear model (glm)	factor	F	p value	summary	Tukey post-test	p value	summary
	Respective	CS A	30	2340	838,2	153	Yes		CS line	13.659	0.0003	***	CS A resp Vs CS B resp	0.0001	***
		CS B	36	1478	706,4	117,7	No		Diet	5.585	0.019	*	CS A switch Vs CS B switch	0.769	ns
	Switch	CS A	24	2138	1013	206,9	Yes		Interaction CS line * Diet	1.304	0.256	ns	CS A resp Vs CS A switch	0.835	ns
		CS B	37	1924	764	125,6	Yes						CS B resp Vs CS B switch	0.062	ns

FIGURE SUPP 4	Diet	CS line	n	Mean	Std. Dev.	S.E.M.	Gaussian distribution	Test					Post-test		
A. Total day slepp								Generalized linear model (glm)	factor	F	p value	summary	Tukey post-test	p value	summary
	Respective	CS A	30	1481	275,1	50,22	No		CS line	25.029	1.902E-06	***	CS A resp Vs CS B resp	0.0002	***
		CS B	36	1711	201,8	33,63	Yes		Diet	0.826	0.365	ns	CS A switch Vs CS B switch	0.016	*
	Switch	CS A	24	1473	192,8	39,36	Yes		Interaction CS line * Diet	0.813	0.369	ns	CS A resp Vs CS A switch	0.999	ns
		CS B	37	1643	206,6	33,97	Yes						CS B resp Vs CS B switch	0.580	ns
B. Number of day sleep bouts								Generalized linear model (glm)	factor	F	p value	summary	Tukey post-test	p value	summary
	Respective	CS A	30	46,93	16,54	3,019	Yes		CS line	3.156	0.078	ns	CS A resp Vs CS B resp	0.765	ns
		CS B	36	50,94	18,28	3,047	Yes		Diet	0.337	0.562	ns	CS A switch Vs CS B switch	0.461	ns
	Switch	CS A	24	48,79	16,67	3,402	Yes		Interaction CS line * Diet	0.999	0.319	ns	CS A resp Vs CS A switch	0.977	ns
		CS B	37	55,35	16,29	2,679	Yes						CS B resp Vs CS B switch	0.701	ns
A. Total night slepp								Generalized linear model (glm)	factor	F	p value	summary	Tukey post-test	p value	summary
	Respective	CS A	30	1834	162,2	29,62	Yes		CS line	4.908	0.028	*	CS A resp Vs CS B resp	0.100	ns
		CS B	36	1915	152	25,33	No		Diet	1.267	0.262	ns	CS A switch Vs CS B switch	0.792	ns
	Switch	CS A	24	1824	113,3	23,13	Yes		Interaction CS line * Diet	1.673	0.198	ns	CS A resp Vs CS A switch	0.993	ns
		CS B	37	1858	136,7	22,47	Yes						CS B resp Vs CS B switch	0.326	ns
B. Number of night sleep bouts								Generalized linear model (glm)	factor	F	p value	summary	Tukey post-test	p value	summary
	Respective	CS A	30	39,07	19,3	3,523	Yes		CS line	6.620	0.011	*	CS A resp Vs CS B resp	0.054	ns
		CS B	36	29,83	11,85	1,975	Yes		Diet	1.070	0.303	ns	CS A switch Vs CS B switch	0.692	ns
	Switch	CS A	24	38,13	14,47	2,953	Yes		Interaction CS line * Diet	0.446	0.505	ns	CS A resp Vs CS A switch	0.996	ns
		CS B	37	33,81	13,49	2,217	Yes						CS B resp Vs CS B switch	0.619	ns
