# Supplementary material for: Long-Term Dietary Restriction Leads to Development of Alternative Fighting Strategies
Source: Front Behav Neurosci. 2021 Jan 14;14:599676. doi: 10.3389/fnbeh.2020.599676 (PMC7840567; doi:10.3389/fnbeh.2020.599676)
Supplement: Supplementary file 9 [file Table_1.docx]

Supplementary Material

**Table Supp 1.** The composition of diets used in this study before and after October 2019, starting point of the behavioral experiments.

**Table Supp 2**. Behavioral patterns used to score aggressive behavior.

**Table Supp 3**. Detailed descriptive statistics and test employed in the main and supplementary figures.

**Figure Supp 1.** Diagram of the experimental design.

**Figure Supp 2**. The latency to wing threat was not different between Cs line (χ²test=1,8, d.f=1, p=0,2) and diet (χ²test=2,6, d.f=1, p=0,1).

**Figure Supp 3. (A)** Winners gave significantly more lunges than did the losers for the 4 groups (χ²test_CSASugar+_=31,36, d.f= 1, p=0,0001; χ²test_CsBSugar_-=96,04, d.f= 1, p=0,0001; χ²test_CsASugar-_=49, d.f=1, p=0,0001; χ²test_CsBSugar+_=81, d.f=1, p=0,0001); (). **(B)** Winners displayed significantly more wing threats than did the losers for the 4 groups (χ²test_CsASugar+_=21,16, d.f=1, p=0,0001; χ²test_CsBSugar-_=96,04, d.f=1, p=0,0001; χ²test_CsASugar-_=54,76, d.f=1, p=0,0001; χ²test_CsBSugar+_=60,84, d.f= 1, p=0,0001).

# Figure Supp 4. (A) A significant difference of activity was observed between CS lines (F=(1,125)=17,62, p=5,11e-05), but not between diet (F=(1,124)=3,70, p=0,056). The post-test indicated only one statistical difference between CS A and CS B when raised on their respective diet. (B) Both factors: CS line (F=(1,125)=5,87, p=0,017) and diet (F=(1,124)=5,22, p=0,024) affected the activity of flies during the night. However, the post-test detected only a statistical difference between CS A and CS B when raised on their respective diet. (C) Both factors: CS line (F=(1,125)=13,66, p=3,3e-04) and diet (F=(1124)=5,58, p= 0,019) affected the activity of flies during the day. But, only a statistical difference was observed between CS A and B when raised on their respective diet.

**Figure Supp 5. (A)** The total amount of sleep during the day was higher in CS B line compared to CS A (F=(1,125)=25,03, p=1,90e-06). Diet did not influence day sleep (F=(1,124)=0,83, p=0,36). **(B)** The number of the sleep bouts during the day was not affected by CS line (F=(1,125)=3,15, p=0,07) nor diet (F=(1,123)=0,33, p=0,56). **(C)** The total amount of sleep during the night was different between CS lines (F=(1,125)=4,90, p=0,028), but no statistical differences were indicated by the post-test. Diet was not a factor influencing this sleep parameter (F=(1,124)=1,27, p=0,26). **(D)** The number of the sleep bouts during the night was different between CS lines (F=(1,125)=6,62, p=0,011), but no statistical differences were obtained with the post-test. Diet did not affect this parameter (F=(1,124)=1,07, p=0,30).
